# Diagnosis of acquired generalized lipodystrophy in a single patient with T-cell lymphoma and no exposure to Metreleptin

**DOI:** 10.1186/s40842-019-0076-9

**Published:** 2019-03-14

**Authors:** Nazanene H. Esfandiari, Melvyn Rubenfire, Adam H. Neidert, Rita Hench, Abdelwahab Jalal Eldin, Rasimcan Meral, Elif A. Oral

**Affiliations:** 1 Division of Metabolism Endocrinology and Diabetes, Department of Internal Medicine, University of Michigan and Brehm Center for Diabetes, 1000 Wall Street, Room 5313, Ann Arbor, MI 48105 USA; 2 Division of Cardiovascular Medicine, Department of Internal Medicine, University of Michigan, Ann Arbor, MI USA

**Keywords:** T-cell lymphoma, Acquired generalized lipodystrophy, Insulin resistance, Diabetes, Leptin

## Abstract

**Background:**

Metreleptin, a recombinant methionyl -human -leptin, was approved to treat patients with generalized lipodystrophy (GL) in February 2014. However, leptin therapy has been associated with the development of lymphoma. We present a unique case of a patient with prior history of T cell lymphoma in remission, who was diagnosed with Acquired Generalized Lipodystrophy (AGL) during the following year after a clinical remission of her lymphoma without receiving leptin therapy.

**Case presentation:**

A 33-year-old woman with a diagnosis of stage IV subcutaneous panniculitis like T-cell lymphoma in 2011, underwent chemotherapy. Shortly after completion therapy, she had a relapse and required more chemotherapy with complete response, followed by allogenic stem cell transplant on June 28, 2012. Since that time, she has been on observation with no evidence of disease recurrence. Subsequent to the treatment, she was found to have high triglycerides, loss of fat tissue from her entire body and diagnosis of diabetes. Constellation of these findings led to the diagnosis of AGL in 2013. Her leptin level was low at 3.4 ng/mL (182 pmol/mL). She is currently not receiving any treatment with Metreleptin for her AGL.

**Conclusions:**

Causal association between exogenous leptin therapy and T-cell lymphoma still remains unclear. We hereby present a case of a young woman who was diagnosed with AGL after going into remission from T-cell lymphoma and who has never been treated with Metreleptin. Steroid therapy and chemotherapy might have masked the diagnosis of AGL in this patient. We believe that patients can develop these 2 conditions independent of each other.

## Background

Lipodystrophy is a rare disorder characterized by selective loss or absence of adipose tissue with leptin deficiency, ectopic lipid deposition, and severe metabolic abnormalities [1]. Lipodystrophy can be congenital or acquired and its distribution can be generalized or partial [2–4]. Metreleptin, a recombinant methionyl-human-leptin, was approved to treat generalized lipodystrophy in February 2014 [5]. Patients with acquired generalized lipodystrophy (AGL) present with loss of adipose tissue, hypertriglyceridemia, severe insulin resistance leading to diabetes and hepatic steatosis [3, 4]. Leptin therapy will improve many of the metabolic conditions in patients with AGL [6, 7]. Many patients with AGL have abnormal immune function and can even develop immunologic malignancies such as T-cell lymphoma [8].

Since a few patients with AGL developed T-cell lymphomas after being exposed to Metreleptin for some time, the FDA placed a black box warning to monitor for lymphomas and immune abnormalities while on Metreleptin even though a causal association between drug exposure and the development of the T-cell lymphoma was not established. In this report, we present a unique case of a patient with prior history of T cell lymphoma in remission, who was diagnosed with AGL during the following year after a clinical remission of her lymphoma. She has never received Metreleptin therapy. We believe that this report will add to the evidence that T-cell lymphoma and AGL may develop in the same patient independent of the use of Metreleptin or exogenous leptin therapy.

## Case presentation

A 33-year-old woman without a significant past medical history, was diagnosed with stage IV subcutaneous panniculitis like T-cell lymphoma in 2011, complicated by hemophagocytic lymphohistiocytosis. She completed 6 cycles of CHOEP regimen (cyclophosphamide, doxorubicin, etoposide, vincristine and prednisone) in December 2011. Shortly after completion of the therapy, she relapsed and received multiple salvage regimens including gemcitabine/oxaliplatin, bexarotene/dexamethasone and pralatrexate. She was then initiated on ESHAP regimen; a combination of the chemotherapeutic drugs etoposide, methylprednisolone, a high-dose cytarabine and cisplatin in April 2012, achieving complete response; followed by an allogenic stem cell transplant on June 28, 2012. Since that time, she has been on observation with no evidence of disease recurrence. Subsequent to the treatment, she was found to have high triglycerides (230 mg/dL [2.60 mmol/L] (normal range < 150 mg/dL [1.69 mmol/L]) in 2011, and 613 mg/dL [6.93 mmol/L] in 2013) and loss of fat tissue from her entire body with accompanying muscular prominence. She was also diagnosed with diabetes in 12/2013. Constellation of these findings led to the diagnosis of AGL in 2013.

**Fig. 1:**
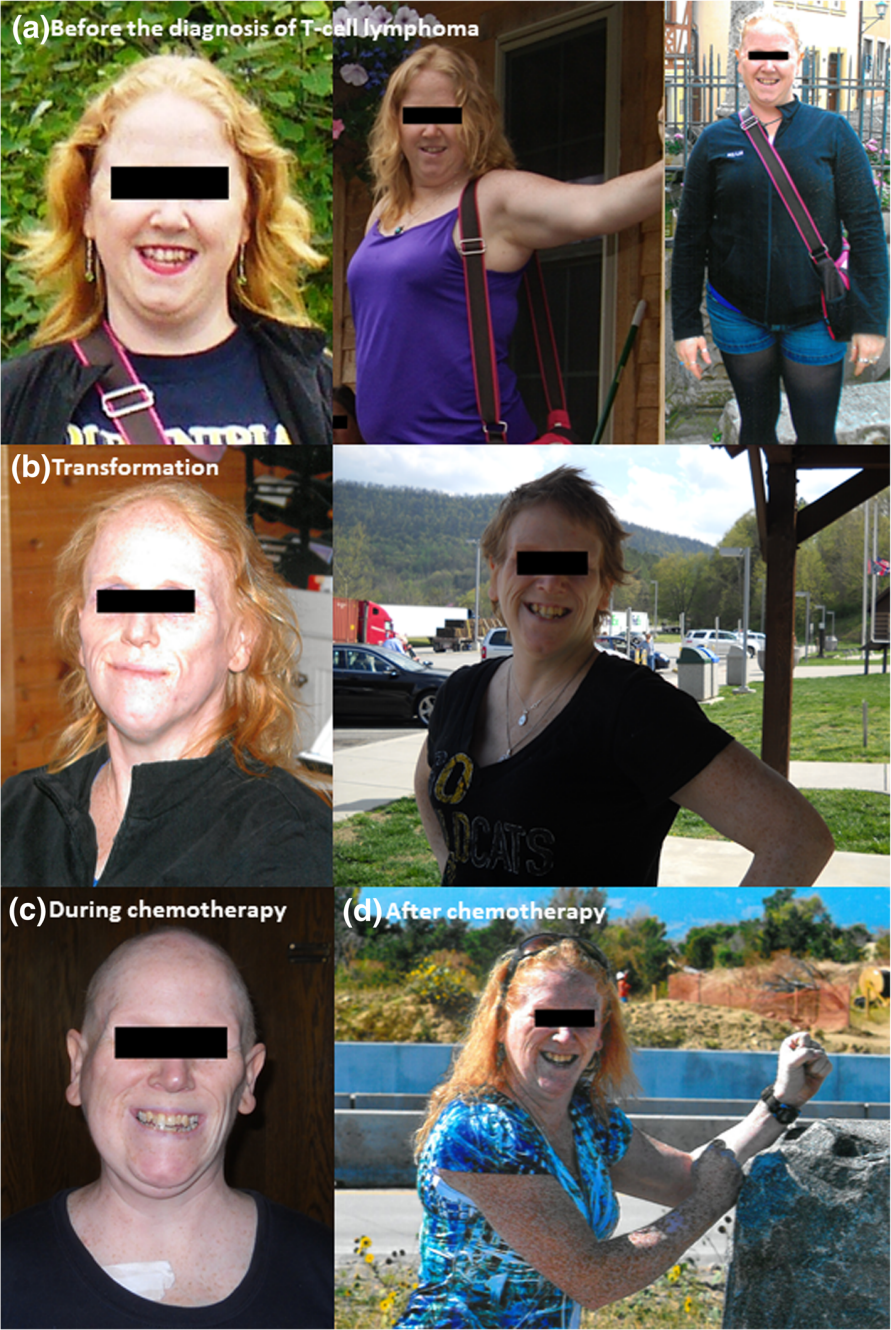
Development of Physical Features of Lipodystrophy. Panel (**a**) shows the patient before the diagnosis of T-cell lymphoma and does not show convincing evidence for generalized lipodystrophy. Physical transformations are shown in panel (**b**) during the diagnosis of the T-cell lymphoma, suggesting that she may have already developed features of lipodystrophy at least around her face and neck. Panel (**c**) shows her appearance during chemotherapy and panel (**d**) is after the chemotherapy. Currently (4 years after the completion of therapy) patient’s appearance is like the picture as shown in (**b**) suggesting that the chemotherapy and steroid use temporarily masked the diagnosis of lipodystrophy and were able to modify the physical appearance

At her visit in our clinic, her BMI was 24.4 kg/m^2^. Her leptin level was low at 3.4 ng/mL (182 pmol/mL) (a level of < 4 ng/mL [215 pmol/mL] is accepted as low for women with BMI < 25 kg/m^2^ despite a wider range provided by some reference labs). Upon obtaining a detailed history, and reviewing her old pictures, it was confirmed that the onset of body fat loss occurred prior to her T-cell lymphoma diagnosis. She also endorsed complaints of hyperphagia and a marked increase in her appetite. Current clinical endocrine problems outside of diabetes and dyslipidemia include increased appetite and hyperphagia, lack of menses and a generalized pain syndrome likely attributable to small fiber neuropathy due to hypertriglyceridemia [9]. Table 1 summarizes her laboratory findings since the diagnosis until her last visit in our clinic. Figure 1 panels a-d show her pictures; before diagnosis (a), at diagnosis of T-cell lymphoma(b), during chemotherapy (c) and after chemotherapy (d).

**Table 1:** Laboratory data from our case

Year	Glucose mg/dL (mmol/L)	A1C % (mmol/mol)	Triglycerides mg/dL (mmol/L)	Creatinine mg/dL (μmol/L)	24-H urine protein
2011	82 (4.55)		230 (2.60)	0.4 (35.4)	0.61^a^
2012	114 (6.33)		416 (4.70)	0.8 (70.7)	
2013	114 (6.33)	6.1 (43)	466 (5.27) 613 (6.93)	1.1 (97.2)	
2014	115 (6.38)	6.4 (46)	1027 (11.6) 2577 (29.1)	0.9 (79.6)	< 5^b^
2015	201 (11.2)	5.9 (41) 7.2 (55)	1372 (15.5) 776 (8.77)	1.05 (92.8)	283^b^
2016	201 (11.2)	6.2 (44) 6.9 (52)	478 (5.40) 1392 (15.7) 3615 (40.9)	1.0 (88.4)	
2017	146 (8.10)		1604 (18.1) 740 (8.36)	1.06 (93.7)	
2018	103 (5.72)	7.7 (61)	4380 (49.5) 336 (3.80)	1.05 (92.8)	

A1C: hemoglobin A1C

^a^and ^b^: reference range for 24-h urine protein in our lab (0–0.15 g/24 h) and in an outside lab (42–225 mg/24 h), respectively

**Fig. 2:**
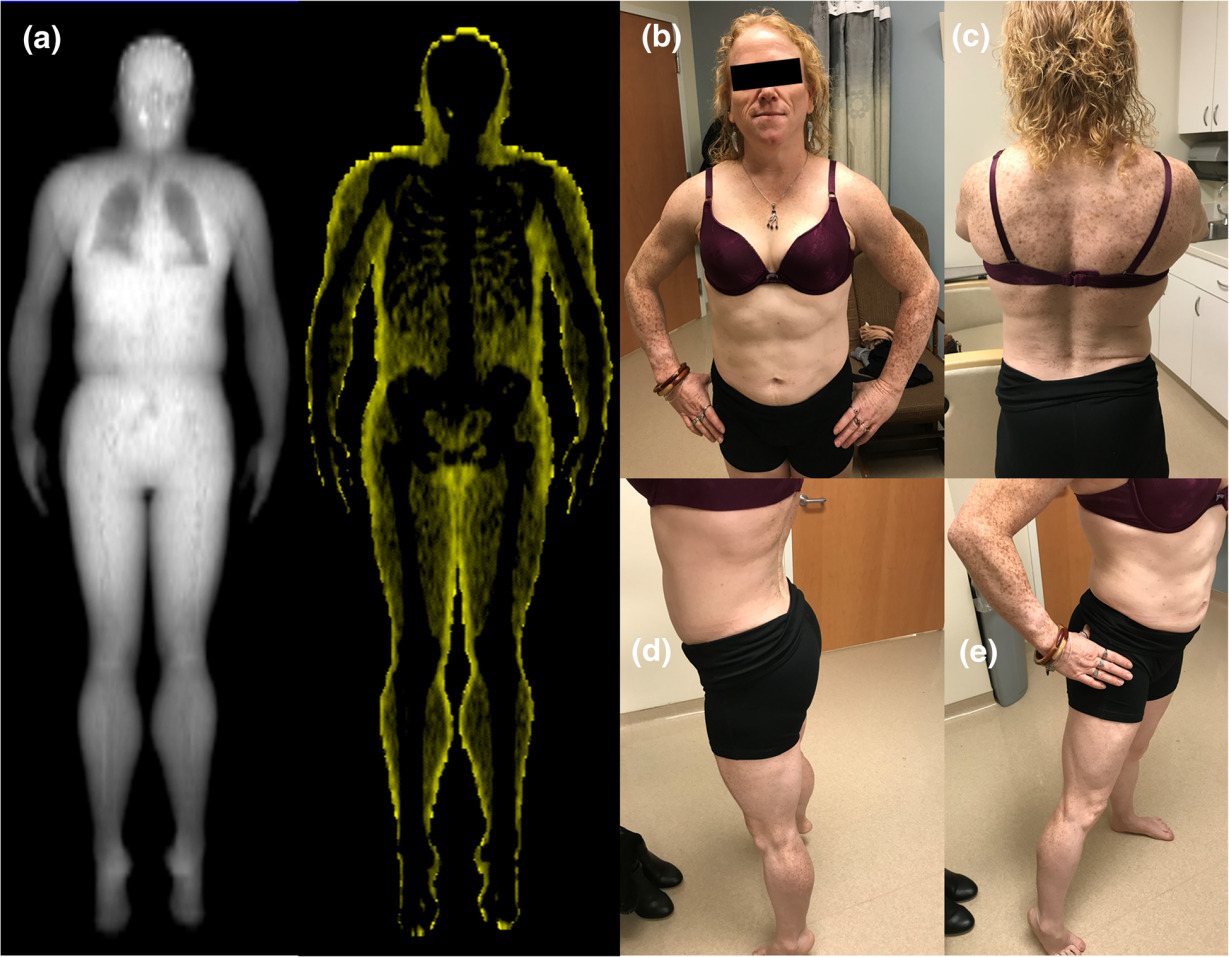
Clinical Examination of Fat Distribution. Panel (**a**) show the “Fat Shadow” obtained from Dual X-ray Energy absorbtiometry (DXA) scan as described previously in Reference [10]. The fat shadow shows minimally retained fat around the neck and axilla, but loss of fat in a generalized fashion though not totally absent from the body. Close-up pictures taken in clinic setting demonstrate fat loss in the face and trunk (**b**), back (**c**), buttock and back of the legs (**d**), and forearms and legs (**e**). The absence of fat from the face together with the entire abdomen, trunk and extremities favor the diagnosis of generalized lipodystrophy as opposed to partial lipodystrophy. Neck fat is preserved, but not excessive. Mons pubis fat was not increased

At her most recent visit, she was doing well and is in remission for T cell Lymphoma. A “fat shadow” [10] representation from her DXA scan (panel a) and her new clinical pictures (panels b-e) are presented in Fig. 2. For her metabolic complications of her AGL, she is currently not being treated with Metreleptin and this possibility is still under discussion. She is on multiple medications to treat her diabetes, high triglycerides and has been trying to limit her food intake.

## Discussion and conclusions

Several cases of T-cell lymphomas after Metreleptin exposure in patients with AGL have been described and led to a black box warning for the approval of Metreleptin in the United States. Over the past several years, there have been several case reports of AGL patients presenting with T-cell lymphomas who have never received leptin therapy. Given the rarity of the AGL, documentation of cases with T-cell lymphoma in the absence of Metreleptin therapy is quite important to provide the evidence base needed to provide assurance that Metreleptin is not playing a causal role in the development of the T-cell lymphomas. Our current report describes a unique case of a patient who developed adult onset AGL (which is quite rare), and who got diagnosed with the AGL after the remission of her T-cell lymphoma. It is important to underscore that this is distinctive than cases of children developing partial lipodystrophy who have undergone hematopoietic stem cell transplantation during childhood [11].

Lymphomas, particularly peripheral T-cell lymphomas (PTCL), have been noted in AGL [12, 13]. Brown et al. reported 5 cases of AGL and lymphoma [12]. The coexistence of AGL and lymphoma likely relates to an underlying autoimmune preponderance. Autoimmune diseases can occur commonly in patients with AGL [8, 12, 14, 15], including both organ-specific autoimmunity (e.g. type 1 diabetes, autoimmune hepatitis) and systemic autoimmune diseases (e.g. juvenile dermatomyositis) [12].

Lymphoma can be considered a systemic feature of AGL instead of an association with lipodystrophy per se given that other forms of lipodystrophy have not been associated with an increased risk of lymphomas [12]. Furthermore, it appears that patients with AGL are at an increased risk of the development of T-cell lymphomas, especially (PTCL), which is a heterogeneous and generally aggressive disorder. It is thought to be related to acquired lipodystrophy, as a subtype of PTCL localizes to the subcutaneous fat, termed subcutaneous panniculitis-like T-cell lymphoma. Panniculitis may be the presenting feature of AGL, as in the patient with AGL and PTCL reported by Yiannias and colleagues [16]. The clinical assessment of the potential role of Metreleptin in contributing to development and/or progression of lymphoma is limited by the lack of long term, controlled studies. Three out of 17 patients with AGL (18%) developed T-cell lymphoma within the NIH cohort [5], an incidence significantly higher than what is observed in the general population (approximately 2 per 100,000) [17].

Another interesting case of PTCL subsequently complicated by AGL has been described by Aslam et al. [18]. Misra and Garg proposed three classification types for AGL based on the etiology and pathological mechanisms: type 1 represents AGL associated with panniculitis; type 2 represents AGL with accompanying autoimmune diseases, and type 3 represents idiopathic AGL [8]. Aslam et al. felt that their patient developed AGL and likely represented an overlap of both the type 2 and 3 varieties [18]. The classification of AGL is likely not to be as simple, and there are newer case reports that may change or challenge these earlier classifications.

The time course of the development of AGL was interesting in our case. One possibility is that our patient started to develop AGL while she was going through the diagnosis of her T cell lymphoma as she had panniculitis related T-cell lymphoma. Her treatment with chemotherapy and steroids may have been a contributing factor in her clinical presentation. Steroids might have also masked the features of AGL until the remission of T cell lymphoma and discontinuation of steroids.

In conclusion, there are several clinical lessons from our case. First, development of extensive panniculitis in an adult patient who is losing body fat should raise suspicion of for both T-cell lymphoma and AGL. These patients should be vigilantly monitored for the development of metabolic complications over time. Finally, the association of T-cell lymphoma with known panniculitis within the setting of AGL may be expected and is not likely impacted by Metreleptin therapy.

## Abbreviations

AGL: Acquired generalized lipodystrophy
GL: Generalized lipodystrophy
PTCL: Peripheral T-cell lymphoma
